# Supplementary material for: Cystic fibrosis swine fail to secrete airway surface liquid in response to inhalation of pathogens
Source: Nat Commun. 2017 Oct 5;8:786. doi: 10.1038/s41467-017-00835-7 (PMC5629252; doi:10.1038/s41467-017-00835-7)
Supplement: Supplementary file 1 — Supplementary Information [file 41467_2017_835_MOESM1_ESM.pdf]

Description of Supplementary Files

File name: Supplementary Information
Description: Supplementary figures and supplementary tables.

File name: Peer review file

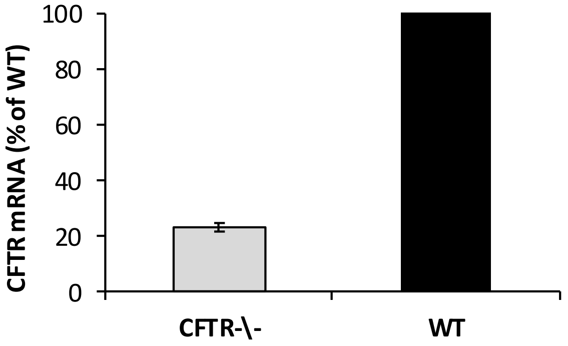

Supplementary Figure 1: CFTR mRNA levels (relative to *β-actin*) in CF gut corrected (CFTR$^{-\backslash-}$) and Wild-type (WT) pigs. Values are shown as percentage mRNA levels relative to the wild-type. Samples were run in quadruplicates. qPCR results showed that CFTR mRNA levels (normalized to *β-actin*) in the gut of pig#7 was 23.29% that of wild-type, which is consistent with what has been previously reported[29].

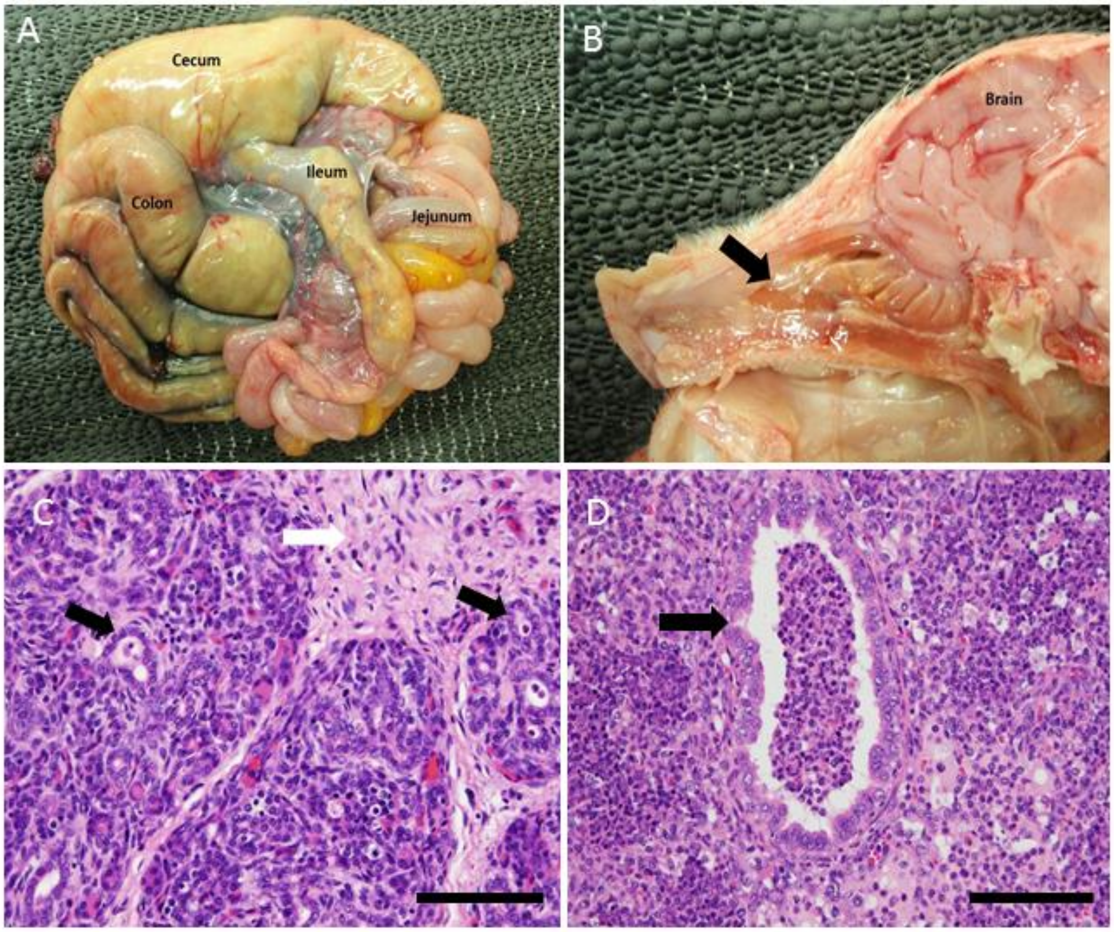

Supplementary Figure 2: Pathology analysis of gut-corrected CFTR$^{-/-}$ swine. The carcasses of 10 animals were subjected to pathology analyses by a board-certified veterinary pathologist. A) Intestines: The cecum and spiral colon are severely distended by feces. B) Head: The nasal cavity contains excessive mucous (arrow). C) Pancreas: The acini (black arrows) are mostly devoid of zymogen granules, and the interstitium contain increase amount of fibrous tissues (white arrows). D) Lung: The bronchiole (arrow) and alveoli are filled with many inflammatory cells, mostly neutrophils. Scale bars indicate 100 μm.

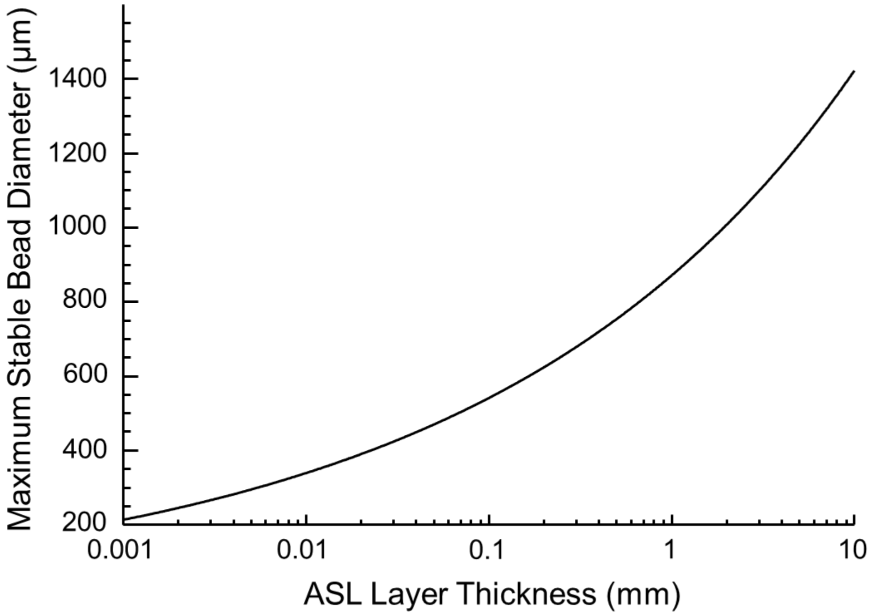

Supplementary Figure 3: Maximum diameter of beads that will be forced against the epithelium at the top of the trachea at a given range of ASL layer thickness. The data shows the change in adhesion force, within the range of ASL thickness (20 to 300 µm) observed in swine trachea, , with varying bead size and fluid thickness as calculated from equation 5.

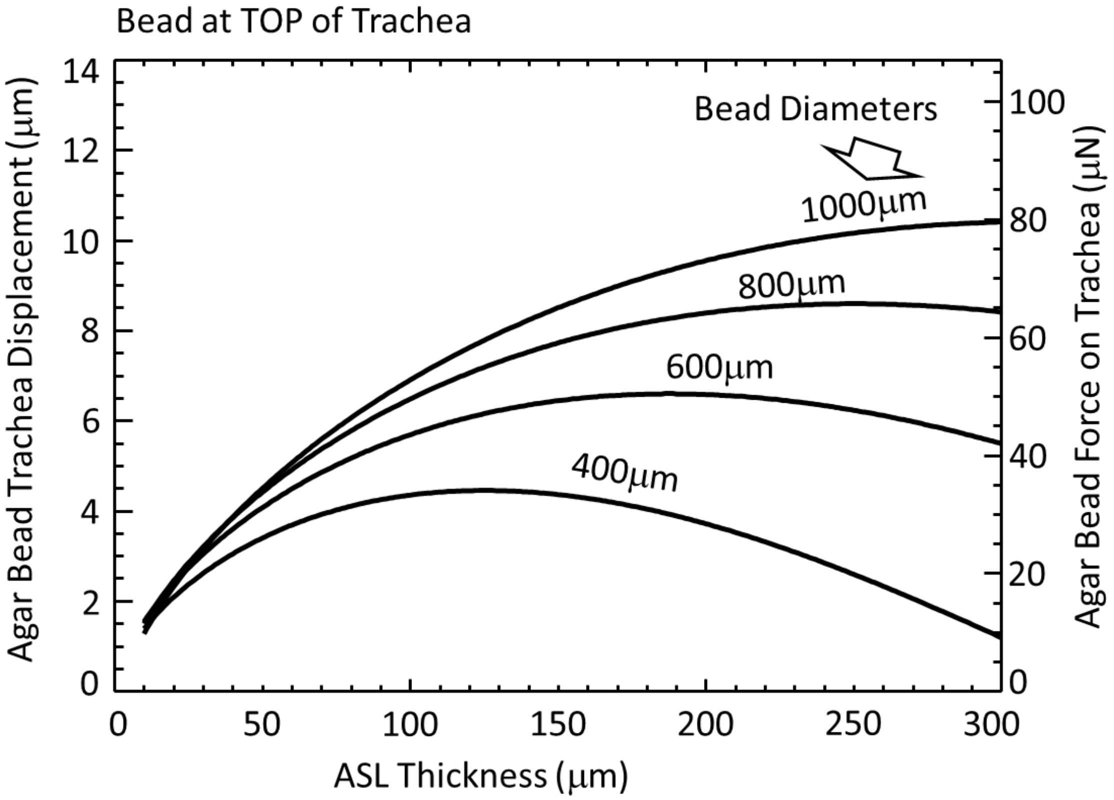

Supplementary Figure 4: Tissue deformation in the top part of the trachea for given ASL thicknesses and bead diameters. The displacement of tracheal epithelium by beads of varying sizes and under varying ASL layer thicknesses can be calculated from equation #6 . Tissue displacement is negligible compared to the height of the ASL layer in live swine trachea.

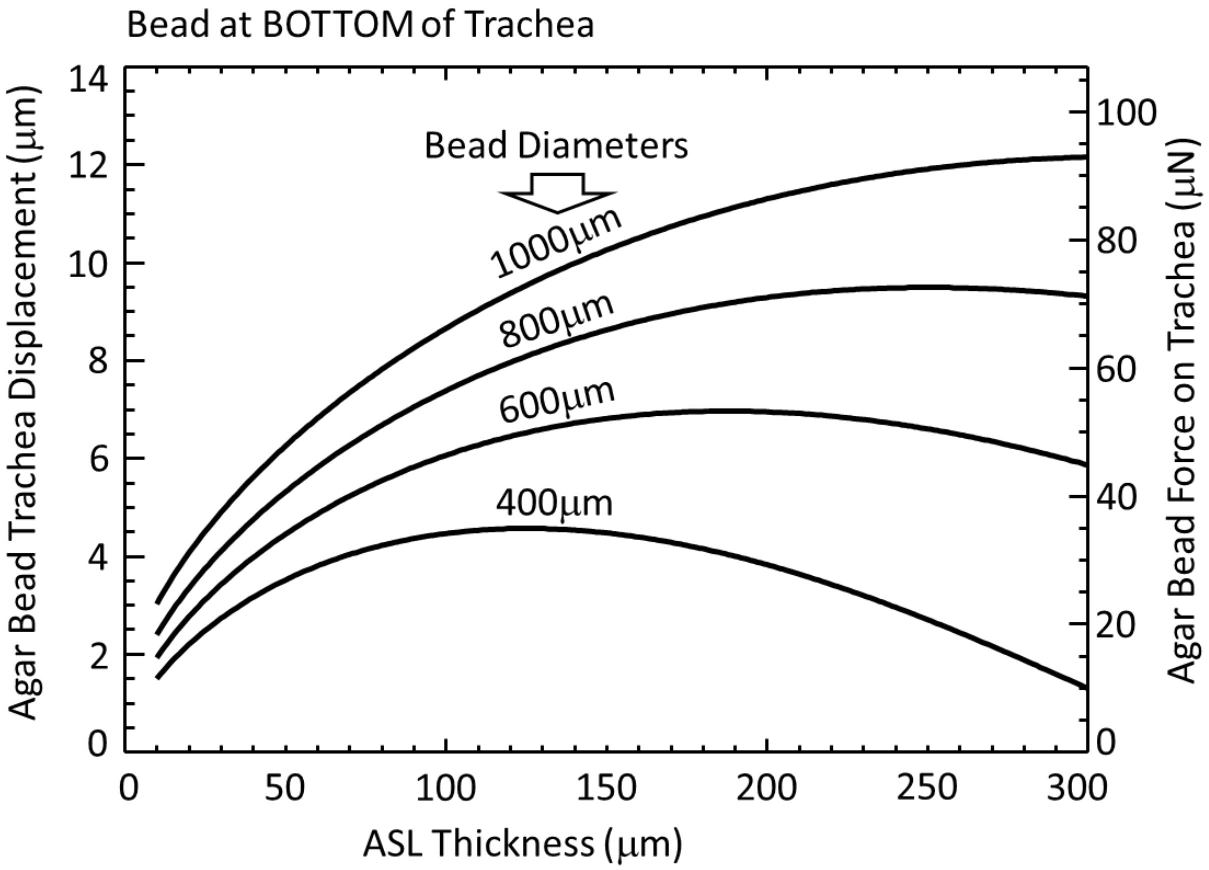

Supplementary Figure 5: Tissue deformation of the bottom part of the trachea for given ASL thicknessnes and bead diameters. The displacement of tracheal epithelium by beads of varying sizes and under varying ASL layer thicknesses can be calculated from equation #6 . Tissue displacement is negligible compared to the height of the ASL layer in live swine trachea.

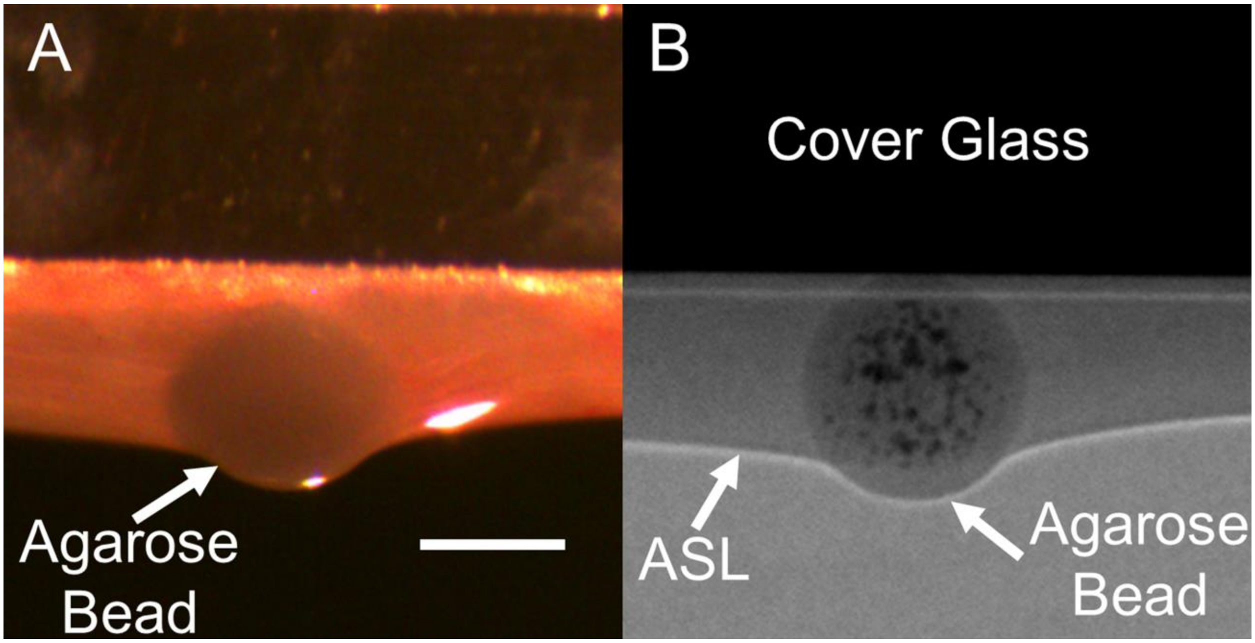

Supplementary Figure 6: Surface tension force the beads against the glass slide. We placed agarose beads in an ASL sample collected from isolated trachea and placed it on the underside of a glass slide. The agarose beads were forced against the glass surface, indicating that the surface tension from the ASL is stronger than gravity and it forces the beads up against the glass surface, as predicted by the mathematical model A) Light, and B) phase contrast images of the same bead immersed in ASL[18]. Scale bar represents 500 μm.

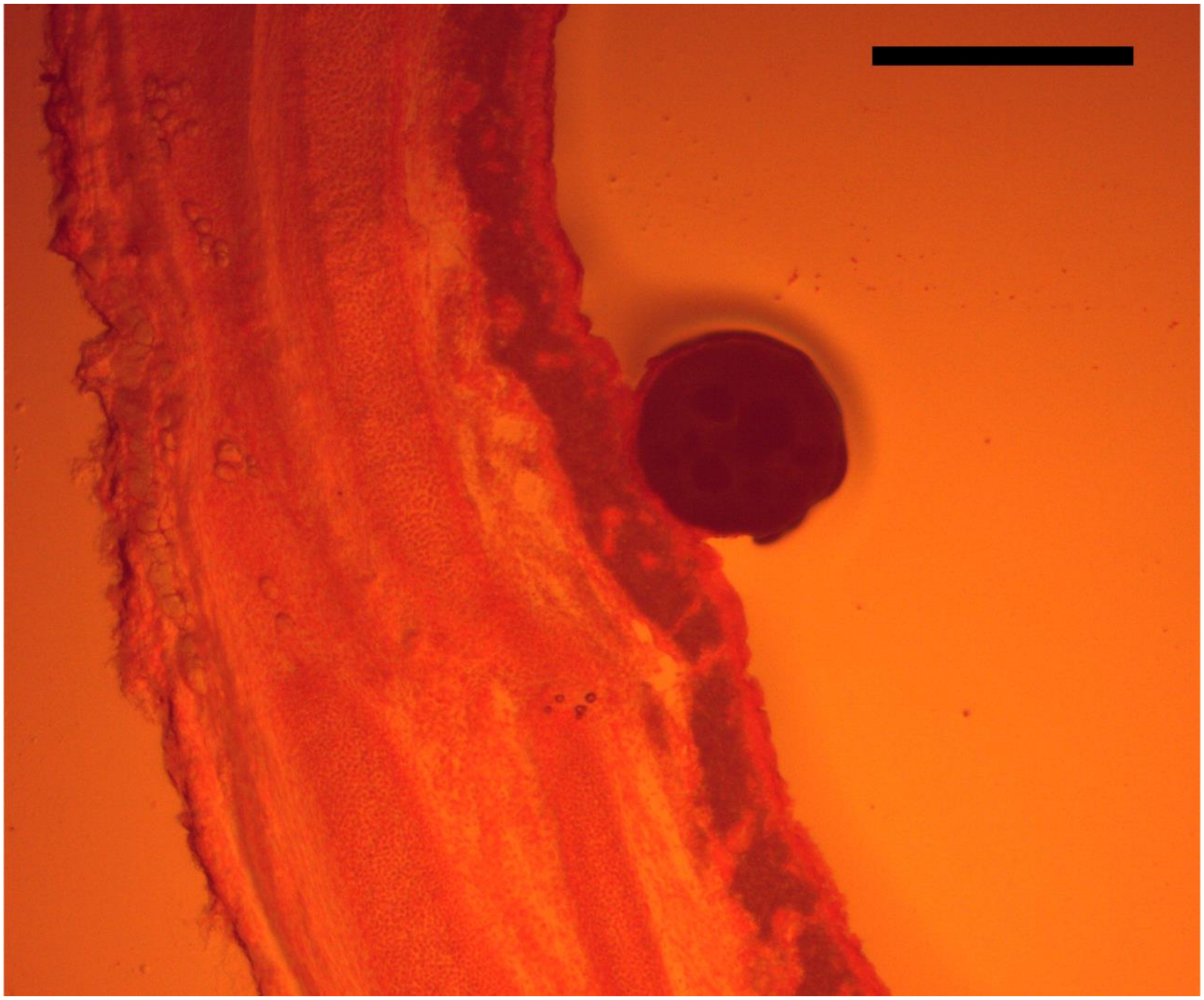

Supplementary Figure 7: Cryo-section of a tracheal preparation showing an agarose bead in contact with airway surface epithelia. Agarose beads were instilled into freshly harvested trachea of 1 week old wild-type piglets. The preparations were flash frozen in liquid nitrogen and stored at -80°C. Then, the trachea preparations were cryo-sectioned into 20 μm sections and stained with Nuclear Fast Red (Sigma) for 1 minute, which delineated the airway tissue. The sections were imaged on a light microscope. Histological images (n=11) clearly showed that the agarose beads were indeed in contact with the airway surface epithelium. Scale bar represents 500 μm.

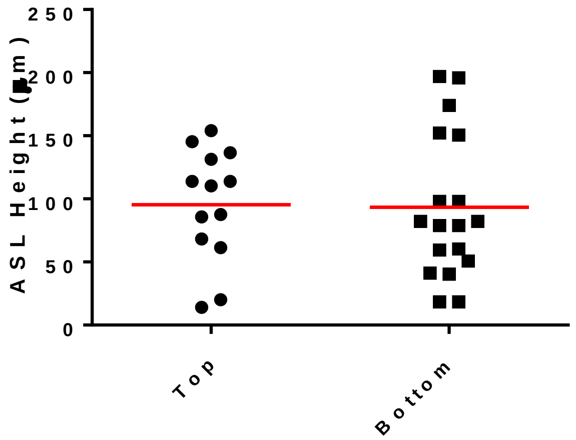

Supplementary Figure 8: Scatterplot and mean (red lines) ASL height measured with beads placed at the top and bottom parts of live swine trachea (n = 13 for top and n = 18 for bottom; t = 0.1239, df = 29, p = 0.90, Students' t-test).

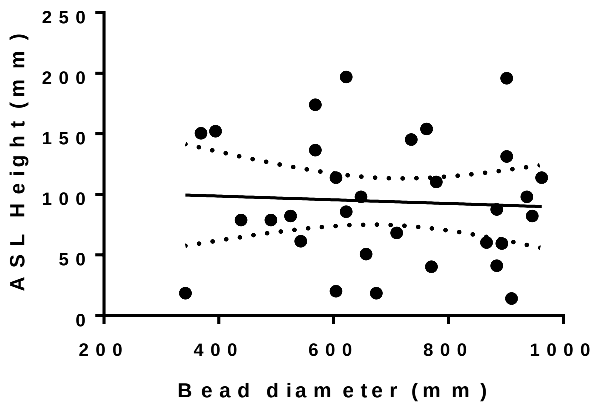

Supplementary Figure 9: Scatterplot and linear regression of bead diameter vs ASL height measured 5 min after incubating tissues with bateria-free beads. The linear regression was not significant (p = 0.77, F = 0.087, DFn = 1, DFd = 29; r^2 = 0.003)

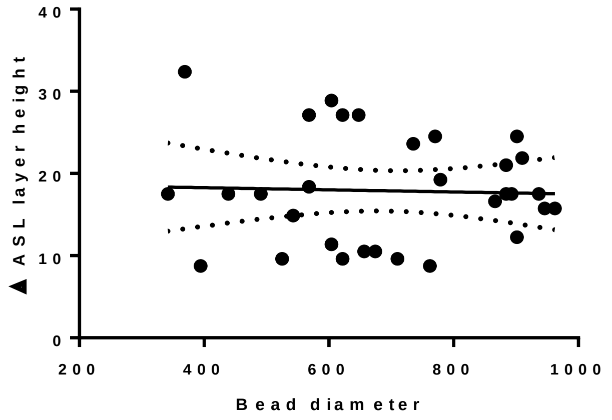

Supplementary Figure 10: Scatterplot and linear regression of bead diameter vs change in ASL height after 30 min of incubation with bacteria-free beads. The linear regression was not significant ($p = 0.84$, $F = 0.038$, DFn = 1, DFd = 29; $r^2 = 0.001$).

Supplementary Table 1: Summary of clinical histories of the gut-corrected CFTR-\- piglets.

**Pig#**	**Vital signs at birth**				**Treatments**	**Euthanasia**	
	Wt	HR	O_2	Temp	some offered more than once	Age	Reason
1	1.13	158	99	37.6	1ml enema (oil)	7 days	elective
2	1.43	172	99	37.8	2.5ml enema (water-oil)	5 days	IC, RS.
3	1.14	149	96	36.7	5% dextrose IP 5cc	2 days	IC
4	1.47	136	96	35.6	5% dextrose IP 5cc	2 days	IC
5	1.18	153	90	36.9	5% dextrose IP 5cc, 2.5ml enema (water-oil)	2 days	IC
6	1.47	176	92	38.2	1ml enema (oil)	5 days	IC, RS.
7	1.25	193	95	37.2	5% dextrose IP 5cc, 2.5ml enema (water-oil)	2 days	RS
8	1	198	88	37.7	2.5ml enema (water-oil)	5 days	IC
9	1.37	170	85	37.8	1.5 ml enema (oil)	7 days	elective
10	1.55	171	93	37.3	2.5ml enema (water-oil)	2 days	IC
11	1.26					12 h	RS

Wt- Weight kg. HR- Heart rate. O_2- % oxygen saturation. Temp- Temperature in degree Celsius. IC- Intestinal complications. RS- Respiratory Symptoms.

Supplementary Table 2: Summary of gross and histological findings

Pig ID	Distended colon and cecum with firmer than normal feces	Hypoplastic gall bladder	Common bile duct blocked by thick bile	Suppurative pneumonia	Notable excessive mucous in nasal cavity	Prominent bile stasis	Pancreatic hypoplasia
1	-	+	-	+	-	-	NA
2	+	+	-	+	-	+	NA
3	NA	NA	NA	NA	NA	NA	NA
4	+	-	-	-	-	-	NA
5	+	+	-	-	-	-	NA
6	+	-	-	+	-	-	NA
7	-	-	-	-	-	-	NA
8	+	+	-	-	+	+	+
9	-	+	-	+	+	-	NA
10	+	-	+	-	-	+	NA
11	+	-	-	-	-	-	+

+: present, **-**: absent, **NA**: observation not available
